# Application of Scenario Analysis and Multiagent Technique in Land-Use Planning: A Case Study on Sanjiang Wetlands

**DOI:** 10.1155/2013/219782

**Published:** 2013-05-30

**Authors:** Huan Yu, Shi-Jun Ni, Bo Kong, Zheng-Wei He, Cheng-Jiang Zhang, Shu-Qing Zhang, Xin Pan, Chao-Xu Xia, Xuan-Qiong Li

**Affiliations:** ^1^College of Earth Sciences, Chengdu University of Technology, Chengdu 610059, China; ^2^Institute of Mountain Hazards and Environment, Chinese Academy of Sciences, Chengdu 610041, China; ^3^Northeast Institute of Geography and Agroecology, Chinese Academy of Sciences, Changchun 130012, China

## Abstract

Land-use planning has triggered debates on social and environmental values, in which two key questions will be faced: one is how to see different planning simulation results instantaneously and apply the results back to interactively assist planning work; the other is how to ensure that the planning simulation result is scientific and accurate. To answer these questions, the objective of this paper is to analyze whether and how a bridge can be built between qualitative and quantitative approaches for land-use planning work and to find out a way to overcome the gap that exists between the ability to construct computer simulation models to aid integrated land-use plan making and the demand for them by planning professionals. The study presented a theoretical framework of land-use planning based on scenario analysis (SA) method and multiagent system (MAS) simulation integration and selected freshwater wetlands in the Sanjiang Plain of China as a case study area. Study results showed that MAS simulation technique emphasizing quantitative process effectively compensated for the SA method emphasizing qualitative process, which realized the organic combination of qualitative and quantitative land-use planning work, and then provided a new idea and method for the land-use planning and sustainable managements of land resources.

## 1. Introduction

As a consequence of global increase of economic and societal prosperity, ecosystems and natural resources have been substantially exploited, degraded, and destroyed in the last century [1–3]. Land is one of the most valuable natural resources because of its close relation with human daily lives, and it is suffering high strength of landscape transformation activities such as mine exploitation, infrastructure construction, and agriculture cultivation, which have an important influence on the composition and quality of land resources [4]. The sustainable management of land resource has become the broadly accepted backdrop for policy and management decisions in most parts of the world [5–8]. Described as an activity that envisages future land arrangements [9], land-use planning has been recognized as a key instrument for identifying and ensuring sustainable land resource uses, improving the livelihoods of rural communities, and thereby achieving sustainable development [10].

Land use/cover change (LUCC) is the result of diverse interactions between society and the environment [11–13]. As such, land-use planning has triggered debates on social and environmental values and on the need for participatory processes to address individual differences in these values [14–19]. Over the past years, a number of efforts were undertaken for land-use planning with the consideration of individual participatory processes. For example, Ishii et al. proposed a new needs analysis method for the conceptual land-use planning of contaminated sites and illustrated this method with a case study of an illegal dumping site for hazardous waste [20]. Helbron et al. presented the use of indicators in a site-specific assessment method for strategic environmental assessment in regional land-use planning [21]. Koschke et al. presented a multicriteria assessment framework for the qualitative estimation of regional potentials to provide ecosystem services as a prerequisite to support regional development planning [2]. Lestrelin et al. examined the extent to which the evolution of Laos' village land-use planning has resulted in increased local participation and improved livelihoods [17]. Fitzsimons et al. aimed to create a quantitative, community-engaged basis for the evolution of multiple land uses [22]. Lagabrielle et al. considered participatory modelling to integrate biodiversity conservation into land-use planning and to facilitate the incorporation of ecological knowledge into public decision making for spatial planning [23]. Magigi and Drescher analyzed local communities' involvement in land-use planning to regulate land use change and customary land tenure challenges in a rapidly expanding city in Tanzania [24]. Those researches show that collaborative planning has become an increasingly popular approach in land-use decision making, particularly in situations where there are multiple actors with conflicting interests. 

Over the last couple of decades, scenario analysis (SA) has become a broadly used tool to provide support and advice to policy makers [25]. In decision-making processes, scenarios can help the decision makers to anticipate possible or potential strategies according to different plausible scenarios, which is usually designed to identify a set of possible futures, where the occurrence of each is plausible, although not assured and not necessarily probable [26]. In this way, SA can be seen as a process of understanding, analyzing, and describing the behavior of complex systems consistently and completely. This kind of systematic analysis is crucial in collaborative planning, and it is widely used in land-use planning [27–33]. These approaches are mainly based on the elicitation of information from a set of people, or a panel of experts or stakeholders, and they are therefore characterized by a high level of subjectivity. Indeed, the quality and performance of SA as a basis for decision support become critically dependent on the quality and performance of the assessments expressed throughout the entire land-use planning process [34]. This represents the primary limitation of such qualitative approaches, particularly when the dynamic complexity of coupled systems is not well understood. Then different planners may get completely diverse planning results, and their scientific creditability has frequently been questioned. Hence, a land-use planning methodology based on a systems approach involving realistic computational modeling and metaheuristic optimization is still lacking in many current practices [35]. Through the above analysis, two key questions will be faced during land-use planning process: one is how to see different planning simulation results instantaneously and apply the results back to assist planning work interactively; the other is how to ensure that the planning simulation result is scientific and accurate.

Landscape digital reconstruction and spatial-temporal distribution simulation based on multiperiod regional land cover data can help to understand the mechanisms and laws of land use succession, recognize the relationship between human activities and land-use changes, predict the future trend of the land use, and ultimately provide strategies to decision maker for land-use planning. A multiagent system (MAS) can be defined as a set of agents that interact in a common environment, able to modify their attributes and their environment [36]. MAS may increase understanding of complex coupled social-ecological systems [37], more particularly in the context of land-use planning [38–41]. The MAS technique can simulate the different planning results based on a systems approach involving realistic computational modeling, so as to provide reference for planning work. More importantly, the data mining technique will be used in the process of multiagent simulation, which ensures the planning process can conform to various regions and get a more accurate, more scientific planning result.

The objective of this paper is to analyze whether and how a bridge can be built between qualitative and quantitative approaches for land-use planning work and to find out a way to overcome the gap that exists between the ability to construct computer simulation models to aid integrated land-use plan making and the demand for them by planning professionals. The specific topic is the integration of scenario analysis with the multiagent system technique, with its application in land-use planning process.

## 2. Theoretical Framework

MAS simulation and SA method both emphasize the role of human factors in the regional land use change and outstand human intervention in the simulation and planning process. This provides the theoretical basis for the combination of two methods. SA method aims to separate uncertain factors and establish system variables, which focuses on qualitative or qualitative and quantitative combination analysis process. The MAS simulation emphasizes the laws of landscape spatial-temporal distribution under influences of various geographic, economic, and other factors, which focuses on quantitative or quantitative and qualitative combination analysis process. This simulation process is based on actual multiperiod land cover data to obtain the transformation rules, which can reflect the regional actual change situations more effectively.

**Figure 1:**
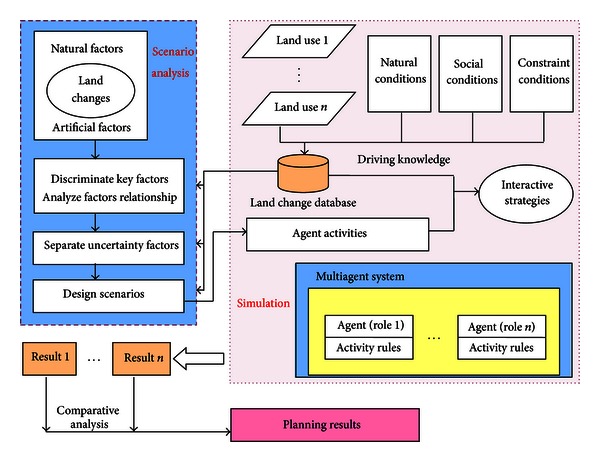
The theoretical framework of landscape planning based on MAS and SA integration.

Through the analysis of MAS simulation and SA method basic principles, combination of them is mainly reflected in the qualitative analysis and design for different scenarios using SA, and actual simulation model and process are completed by MAS quantitatively. Furthermore, factors analysis in scenario design process will be affected by the data mining results of landscape dynamic knowledge database in the MAS simulation process. This makes the scenario analysis process more consistent with the actual situation of regional development and gain a strong geographical significance. The study presents a theoretical framework of land-use planning based on SA method and MAS simulation integration (Figure 1).

## 3. Case Implementation and Results

### 3.1. Study Area and Data

#### 3.1.1. Study Area

**Figure 2:**
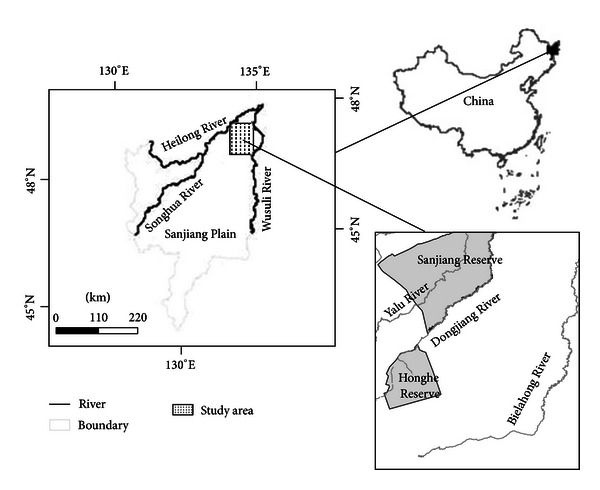
Location of the study area in Sanjiang Plain, China.

Wetlands are integral parts of the global ecosystem as they can prevent or reduce the severity of floods, feed ground water, and provide unique habitats for flora and fauna [42, 43]. The Sanjiang Plain, located in the Northeastern region of China, is one of the largest freshwater wetland in the country (Figure 2). Since the end of the 1950s, large-scale development in the Sanjiang Plain marsh land has occurred [44]. By 2003, about 80% of natural wetlands had been converted to farm land and the progressive loss of wetland is continuing [45]. With a local population of 7.8 million in this region, of which 53.4% is engaged in farming the Sanjiang Plain has become an important grain and bean production region for China [46]. The regional climate is mild humid to subhumid continental monsoon feature. The average temperatures range from −18°C in January to 21-22°C in July with a frost-free period of 120–140 days. Annual precipitation is somewhere around 500–650 mm with 80% occurring from May to September. Most of the rivers at the area have riparian wetlands supporting meadow and marsh vegetation. Sedge (*Carex* spp.) is the dominant plants with *Phragmites* spp. scattered across some parts of the landscape [47].

The study area is limited within 47°21′42′′–48°15′9′′ north altitude and 133°25′52′′–134°33′37′′ east longitude in the Northeast of Sanjiang Plain at (Figure 2). Several factors had been taken into consideration when this region was chosen to start this study. Firstly, the Sanjiang Plain is one of the largest marsh distribution region. Secondly, it is a typical representation in the global temperate wetland ecosystems. Thirdly, due to the relative cold weather, deep surface waters, large marsh patches, and sparse population, reclamation of marsh lands in this region is relatively late. Fourthly, study area contains two national nature reserves and three major river systems: Honghe Reserve, Sanjiang Reserve, and Yalu River, Dongjiang River, Bielahong River. They make the study area possess natural original scenery relatively. In addition, during the process of development and utilization in recent decades, the conflict between people and land is a constant game of war. Wetland degradation process under the disturbance from human activities is representative, which makes it suitable for carrying out simulation of wetland landscape spatial-temporal evolution.

#### 3.1.2. Data

To complete the simulation using MAS, land-use data were collected during three-year period (1995, 2000, and 2006). The 1995 and 2000 datasets are used in decision ruling on transformation, while 2006 dataset is used to verify predicted results. Each land use data set contains 5 types of covers such as water, farmlands, resident area, forest, and wetlands. The data of soil, topography, terrain, location, and other thematic parameters are sorted to formulate the transformation probability under the influence of many geographical conditions. The soil data represent 22 different types; topography data contains 14 types of landforms; River distance is a grid file that reflects the distance to rivers and road distance reflects the distance to road. The units of river distance, road distance, and digital elevation model (DEM) data are meters, and the slope is degree. In addition, the existing data collections previously include planning, feasibility reports, scientific research reports, maps and documentation of Honghe National Natural Reserve and Sanjiang National Natural Reserve, and meteorology, hydrology, groundwater observations and other statistical records are available for reference in this research. The detail of each dataset is listed in Table 1. All of the data were coregistered and formatted as GRID format under ArcGIS 9.3.

### 3.2. Scenario Analysis and Design

#### 3.2.1. The Key Variables and Their Interactions

There are mainly two kinds of factors influencing land use [48, 49]. The first one is direct, and it consists of various forms of activities including conservation and development. The second one is indirect, and it relies on the legal instruments of public policy to influence the behavior of landowners [50]. 

Considering the actual situation of study area, one direct factor is mainly performed by protectors who are the staff of national nature reserves. They prevent wetlands from developing to other landscape directly. Secondly, the statistic results of land use/cover change cells between the years of 1995 and 2006 show that total area changing from wetland to other cover types is 2402.68 km^2^, among which 2215.75 km^2^ is from wetlands to farmlands. The farmlands count about 92.22% of total altered area, and this indicates that the cultivation is the main factor that results in the wetland shrink. Then, another direct factor is mainly performed by farmers who change the land cover through reclaiming wetland. Thirdly, because local governments plan for and decide current and future land use, their role in land use/cover change is crucial. The behavior of the governments includes the government's macromanagement and policy establishment, which are indirectly influence regional landscape change. At last, three main variables that cause regional land use change can be simplified as protectors, farmers, and governments according to study area actual situations.

**Figure 3:**
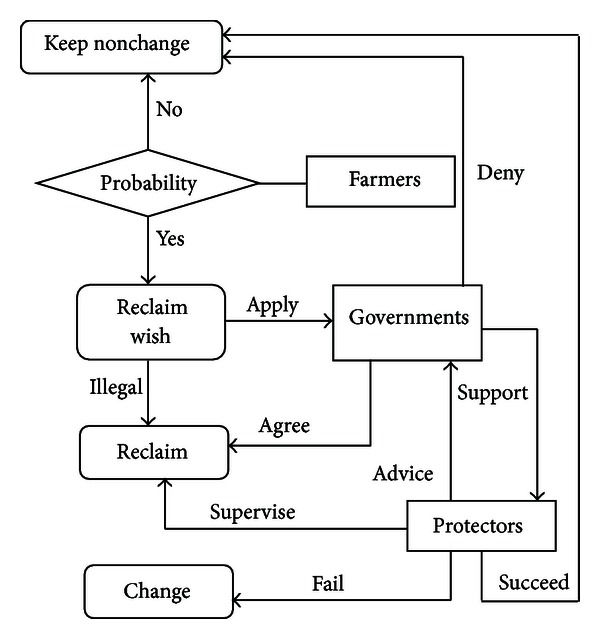
Interaction logistics of variables.

Although one variable has certain functions, however, relying on a single variable cannot always describe and solve complex large-scale problems in reality. Therefore, an application system often includes multiple variables. Each variable is not isolated but an interactive part of the group. Those variables can follow some kind of specific agreement and possess multilinguistic communication skill to complete a specific task. According to the actual situation of study area, logical interaction rules among governments, protectors, and farmers variables are designed as shown in Figure 3.

During the land-use change process, land cover status of certain position is determined by governments, farmers, and protectors variable jointly. First, farmers variable determine whether to reclaim wetlands under various environmental conditions. If the farmers wish to do so, a small part of them will illegally reclaim wetlands, and when this part of farmers goes around obstacles from protectors, the land cover status will be changed. On the contrary, if they are hampered successfully by protectors, then the land cover remains unchanged. The rest of them will apply to the governments variable to reclaim wetlands. Two total diverse consequences will result depending on the government approval to their petition: if the government approved and farmers avoided obstacles from protectors, the land cover status would be changed; if not, the land cover status will be unaffected and unchanged.

#### 3.2.2. The Scenario Design

After finishing the key factors discrimination and interaction relationship analysis, study defined 3 specific planning scenarios in order to verify the availability of theoretical framework presented by this paper.

*Undisturbed Scenario.* According to actual land-use change rules of study region from 1995 to 2000, the land cover of year 2006 will be predicted based on MAS simulation technique.

*Ecotype Scenario.* In this scene, governments' criterions of wetland development approval become strict, and farmers and protectors' awareness of ecological environment protection is strengthened, which cause the reclaim desire to reduce and protection scrutiny to increase. Under these conditions, damage degree of wetland landscape will be degraded. However, it is also very likely to cause regional economy development to be slowed down, and then the income of farmers and governments may drop.

*Economy Scenario.* Governments encourage farmland development for the needs of economic construction, which affects the speed and manner of the entire regional land-use change. Being driven by economic interests, farmers also strongly destroy wetlands for increasing farmland quantity. Protectors abandon wetland conservation efforts and even join in the wetland destruction and agriculture development in action. Under these conditions, the regional land use subordinates the economic construction and ignores the protection of ecological environment. It is a nonsustainable development mode, but a certain degree of economic achievements may arise in this case.

Research realizes different planning scenarios through modifying decision-making behavior of governments, farmers, and protectors variables. It can clearly explain the specific reasons for the differences of diverse planning scenario simulation results. In detail, undisturbed scene is realized through MAS simulation based on the transformation rules that were gained by data mining technique. Governments, protectors and farmers variables are only used to reflect actual land-use change process. An ecotype scenario is realized through governments auditing standards or reducing the rate of approvals, protectors reinforcing supervision to prevent wetlands being destroyed, and famers reducing their cultivation will. On the other side, an economy scenario is realized through which governments lowering standards or increasing the rate of approvals, protectors reducing supervision to increase rate of development, and famers increasing willingness to reclaim wetlands. After finishing the discrimination of key factors, the analysis of their interactions, and the design of scenarios, the next step is how to quantitatively descript them based on MAS computer simulation models.

### 3.3. Model Construction of MAS

#### 3.3.1. Construction of Environmental Factors Layer

Environmental factor layer in the model is the natural and social environment of MAS, the database for land cover spatial-temporal evolution simulation, and a key element of the model [51, 52]. In this model, environmental factor layer is defined as an integral body including the status of initial land cover, elevation, slope, soil, topography, distance to road, distance to river, and other environmental factors.

#### 3.3.2. Definition of Roles and Conduct Rules

A key issue of multiagent model construction is how to abstract and descript agents properly [53]. Analysis of study area land cover reveals the driving force of regional landscape changes that are caused by human activities. Therefore, the simulation of landscape spatial-temporal evolution using MAS is to link up human activities and agents based on multi-agent characteristics. According to scenario analysis and design results, three agent types are defined as, farmer agent, protector agent and government agent.

*Protector Agent. *Protectors who are the staff of national nature reserves in this research prevent farmers from agricultural developing. Protector agent becomes the main driving force slowing down wetland landscape degradation with the support from government agent. The protective efforts of the protector agent are directly reflected on wetland area changes in a specific period of time. The wetland area reduces enormously and quickly, and it is indicative of a poor effect of protection and a small effect on wetland protection. Thus, an equation assessing the protective effect can be quantitatively expressed as

(1) $$\begin{array}{c} P_{\text{omit}}=\frac{A_{\text{marsh}}^{t1}-A_{\text{marsh}}^{t2}}{A_{\text{marsh}}}\times 100\%, \end{array}$$

where *P*_omit_ is the probability that omits hindering agriculture development, *A*_marsh_^*t*1^ is the wetland area at time *t*1, *A*_marsh_^*t*2^ is the wetland area at time *t*2, and *A*_marsh_ is the reserve total area.

Formula (1) will be used to assess the protect effect on Honghe and Sanjiang National Nature Reserves separately. When land cover change position (*i*, *j*) is within those reserves, the omit probability will be calculated using this equation. The omit probability will be one hundred percent if the position is out of the reserves. In such case, protector's activity can be expressed as

(2) $$\begin{array}{c} P_{\text{pro}}\left(i,j\right)=\{\begin{array}{cc} P_{\text{omit}}\left(i,j\right) & \left(\text{within}\;\text{reserves}\right),\\ 1 & \left(\text{out}\;\text{of}\;\text{reserves}\right). \end{array} \end{array}$$

*Farmer Agent. *The behaviors of farmer agent can be classified into two categories: development and undevelopment. This behavior will cause two types of possible results: one is negative behavior that can reduce the wetland area, and the other is a positive activity that does not change the land cover. In reality, farmers perform such activities under the approval of government. In this model, one part of the farmers' behavior is carried out directly (illegal development), while the other part applies for government agent approval selectively. The whole behavior is also affected by protector agent. It must get the approval of government and avoid the hindering effect from protector agent. As a result, the land cover status can be changed eventually.

When the model starts running, the farmers' probability of reclaim wetlands will be calculated by the formula below

**Figure 4:**
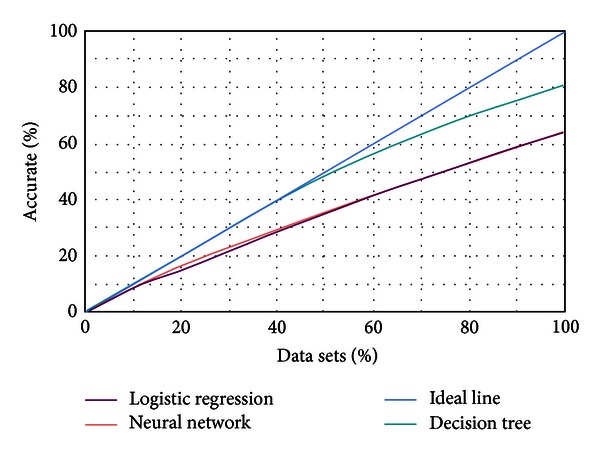
The lift map of data mining.

(3) Pfar(i,j)=(w1Eeleva,w2Eslop,w3Esoil, w4Elandf,w5Erivd,w6Eroad,w7Rdisb),

where *P*_far_(*i*, *j*) is the change probability for position (*i*, *j*); *E*_eleva_, *E*_slop_, *E*_soil_, *E*_landf_, *E*_rivd_, *E*_road_, and *R*_disb_ are the environment factors of elevation, slope, soil, landform, distance to river, distance to road, and random disturbance. These indicators were selected because they are representative of the most critical environmental issues of the study area, and they are easy to understand and communicate. *w*_1_,…, *w*_6_ is the influence weight for each factor, which is calculated through data mining method. This instance completed the calculation of farmland development probability using Microsoft SQL Server 2008 software, data mining process used neural network, decision tree, and logistic regression mathematical model, respectively, and results of them described by lift chart (Figure 4). A lift chart is used for comparing the accuracy of each prediction model. The *x*-axis represents the percentage of the test data set for prediction, and the *y*-axis indicates the percentage of accurate prediction. An ideal line is a diagonal line, which means 50 percent of the data accurately predicted 50 percent of the cases (the expected maximum). Then we found that decision tree method was the best, and then it was used to calculate the probability of land use change under the influence of various geographical conditions.

The random disturbance is expressed as

(4) $$\begin{array}{c} R_{\text{disb}}=1+{\left(-\ln\theta \right)}^{\alpha }, \end{array}$$

where *θ* is the random number between 0 and 1; *α* is the parameter that controls the size of random disturbance; weight *w*_7_ for it is set as 1 [54].

*Government Agent. *Government achieves its own wish through the planning actions, while residents affect the probability of land cover conversion through the cooperation with the government [55]. The behavior of the government agent at the area includes the government's macromanagement, action planning, and decision making in response to farmer agent's application [56]. When farmers apply to convert wetlands for agricultural purpose, government agent will make decisions based on current land cover status and future planning of utilization. And degree of support from government also indirectly determines the specific action of protector. A strongly support will obtain an excellent performance of protection and vice versa. Then the behaviors of the government agent can be expressed through the activities of farmer and protector indirectly as

(5) $$\begin{array}{c} P_{\text{gov}}\left(i,j\right)=\left|1-\left(aP_{\text{pro}}\left(i,j\right)+bP_{\text{far}}\left(i,j\right)\right)\right|, \end{array}$$

where *P*_gov_(*i*, *j*) is the probability that government approves to change land cover for position (*i*, *j*); *a* is the adjustment coefficient for protector between 0 and 1; *b* is the adjustment coefficient for famers between 0 and 1; coefficients are varied with the government policy changing. 

Different scenarios are realized through revising 3 kinds of agents, and the details are shown in Table 2.

### 3.4. Simulation Results

**Figure 5:**
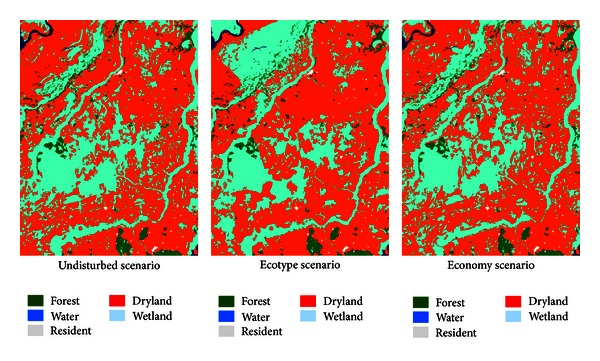
The results of scenarios planning based on multi-agent adjustment.

Based on the characteristics of three planning scenarios, combined with the framework of interaction relationships between governments, protectors, and farmers variables (Figure 4), MAS model is used to generate the different planning scenarios (Figure 5).

### 3.5. Assessment of Accuracy

**Figure 6:**
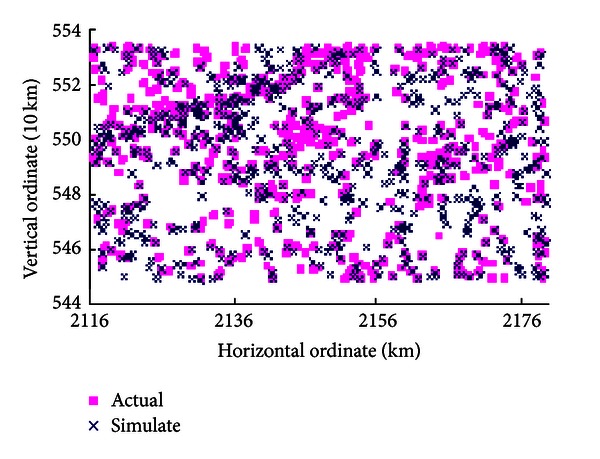
The spatial distribution of centroids.

In order to facilitate observation of the differences between simulation results and reality situations, centroids of each landscape patch including undisturbed scenario simulation results and reality situations of year 2006 are calculated. The spatial distribution of them is plotted, which is shown in Figure 6.

The centroids distribution plots show that simulation results of undisturbed scenario have a high degree of consistency with 2006 year's actual situation. For the quantitative evaluation of the simulation accuracy, study overlaps the simulation result map of undisturbed scenario and the actual land cover map of 2006 together to gain the simulation accuracy of points to points. The overall simulation accuracy of undisturbed scenario reaches 85.12%, and it validates the feasibility and effectiveness of land cover change simulation using MAS. At the same time, it assures the scientific creditability of the other two planning scenarios' simulation results based on SA method and MAS technique integration.

## 4. Discussions and Conclusions

Simulation results show that three different scenarios show vast differences, especially that the eco-type scenario is significantly different from the other two scenarios, which means that the current ecological conditions of the study area are not ideal, and that adjustment and optimization work should promptly be carried out to protect wetlands. The differences between economy scenario and undisturbed scenario are relatively small, but obvious differences still exist on closer inspection. Wetland patches distribution is loose in economy scenario, and many ecologically significant wetland patches are disappeared.

According to the simulation results of different scenarios, depending on the development goals, different planning strategies can be gained. Furthermore, the similarities and differences between actual situations and simulation scenarios can be used to assist land use optimization problem, even to provide reference for landscape reconfiguration including wetland sustainable development, returning farmland to wetland, and so forth.

To sum up, a theoretical framework was proposed for the land-use planning based on the integration SA method with MAS simulation. Taking Sanjiang Plain inland freshwater wetland areas as an example, the study verified the availability of this framework. Results showed that MAS simulation technique emphasizing quantitative process effectively compensated for the SA method emphasizing qualitative process, which realized the organic combination of qualitative and quantitative land-use planning work and then provided a new idea and method for the land-use planning and sustainable managements of land resources.

Applying SA method originated in enterprise management and MAS technique from the field of artificial intelligence into the complex geographical system problem, there is still a lot of refinement work that needs to be further completed. How to establish criteria for the classification of different planning scenarios in SA more scientifically based on regional characteristics and how to define the simulation variables and their interaction relationships based on land-use planning objective more accurately still need to be explored in further practical work.

## Figures and Tables

**Table 1:** List of data description.

Name	Content	Resolution	Time	Source	Size
Soil	Spatial distribution of soil types	30 m	1985	Digitizing	8.41 MB
Landform	Spatial distribution of geomorphologic types	30 m	1985	Digitizing	8.41 MB
River distance	Distance to rivers	30 m	1998	Euclidean distance calculation	33.66 MB
Road distance	Distance to roads	30 m	1998	Euclidean distance calculation	33.66 MB
DEM	Digital elevation model	30 m	1986	Digitizing	33.66 MB
Slope	Spatial distribution of slope	30 m	1986	Calculated from DEM	33.66 MB
Land use	Spatial distribution of land cover types	30 m	1995	TM image classification	16.82 MB
Land use	Spatial distribution of land cover types	30 m	2000	TM image classification	16.82 MB
Land use	Spatial distribution of land cover types	30 m	2006	TM image classification	16.82 MB

**Table 2:** Realization of scenarios through agent parameter adjusting.

Scenarios	Protectors	Famers	Governments	Illustrations
Undisturbed	*P*_pro_	*P*_far_	*P*_gov_	All the parameters keep unchanged
Eco-type	1/3∗*P*_pro_	1/3∗*P*_far_	1/3∗*P*_gov_	Governments reduce the rate of approvals, protectors reinforce supervision, and famers reduce their cultivation will.
Economy	3∗*P*_pro_	3∗*P*_far_	3∗*P*_gov_	Governments increase rate of approvals, protectors reduce supervision, and famers increase willingness to reclaim.
